# Brevetoxin and Conotoxin Interactions with Single-Domain Voltage-Gated Sodium Channels from a Diatom and Coccolithophore

**DOI:** 10.3390/md19030140

**Published:** 2021-03-02

**Authors:** Ping Yates, Julie A. Koester, Alison R. Taylor

**Affiliations:** Department of Biology and Marine Biology, University of North Carolina Wilmington, Wilmington, NC 28403, USA; pngyates17@gmail.com (P.Y.); koesterj@uncw.edu (J.A.K.)

**Keywords:** phytoplankton, membrane excitability, diatom, coccolithophore, algal toxin, brevetoxin, µ-conotoxin

## Abstract

The recently characterized single-domain voltage-gated ion channels from eukaryotic protists (EukCats) provide an array of novel channel proteins upon which to test the pharmacology of both clinically and environmentally relevant marine toxins. Here, we examined the effects of the hydrophilic µ-CTx PIIIA and the lipophilic brevetoxins PbTx-2 and PbTx-3 on heterologously expressed EukCat ion channels from a marine diatom and coccolithophore. Surprisingly, none of the toxins inhibited the peak currents evoked by the two EukCats tested. The lack of homology in the outer pore elements of the channel may disrupt the binding of µ-CTx PIIIA, while major structural differences between mammalian sodium channels and the C-terminal domains of the EukCats may diminish interactions with the brevetoxins. However, all three toxins produced significant negative shifts in the voltage dependence of activation and steady state inactivation, suggesting alternative and state-dependent binding conformations that potentially lead to changes in the excitability of the phytoplankton themselves.

## 1. Introduction

**Figure 1:**
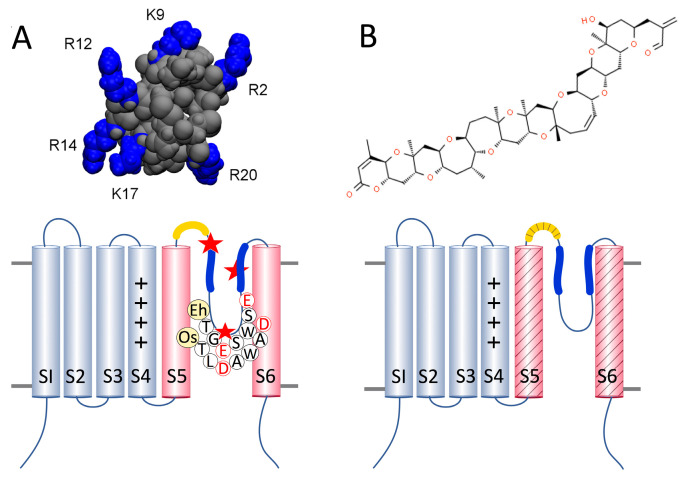
Structure of toxins and their hypothesized binding sites on one subunit of a single-domain channel. (**A**) upper: µ-CTx PIIIA with six positively charged arginine (R) and lysine (K) residues highlighted in blue, from Chen et al. 2014; lower: diagram of a 1D Na_V_/Ca_V_ with the voltage sensor of transmembrane segments S1–S4 (grey) and the pore-domain (pink) consisting of S5, the turret loop (yellow), the P1 and P2 helices (blue) with the selectivity filter sequences of EhEUKCATB1 and OsEUKCATA1, and S6. The red stars represent negatively charged acidic residues aspartic (E) or glutamic (D) acid binding sites near P1 and the selectivity filter [8] and the space between P2 helices of adjacent subunits that µ-CTx PIIIA may occupy when channels are inactivated [9]. (**B**) upper: PbTx-2; lower: regions of PbTx binding across S5, the turret loop, and S6 are represented by the diagonal shading based on homologous regions of binding in sensitive Na_V_s, Na_V_1.2, and Na_V_1.4 [10,11].

Membrane excitability in phytoplankton is facilitated by voltage-gated ion currents similar to those observed in multicellular organisms [1,2]. Many species encode four-domain (4D), animal-like sodium/calcium channels (Na_V_/Ca_V_) in addition to the single-domain (1D) channels recently characterized as the EukCats [3,4]. EukCats are widespread among marine protist groups, including diatoms, coccolithophores and dinoflagellates [3], and contribute to the generation of fast action potentials that play a role in environmental sensing [2,3]. EukCats are homologs of 1D bacterial sodium channels (BacNa_V_s) [3,5] that putatively form functional voltage-gated ion channels from homotetramers. Each 1D monomer of the EukCats contains six transmembrane segments (S1–S6), a voltage-sensing domain (VSD) from S1–S4, with four conserved, positively charged residues on S4, and a selectivity filter (SF) within the pore-loop between S5 and S6. The SF is linked to S5 and S6 by two helices, P1 and P2, respectively (Figure 1). Due to the newfound diversity of 1D channels, we are no longer able to predict ion selectivity solely based on the number and identity of negatively charged residues in the primary sequence of amino acids in the seven-residue selectivity filter. For example, the bacterial 1D NaChBac (*Bacillus halodurans*) and Na_V_Pp (*Plesiocystis pacifica*) channels are both Na^+^-selective, but they have very different selectivity filters [6,7]. NaChBac contains only a single negatively charged glutamic acid (E) in its SF (TLESWAS) [6], whereas Na_V_Pp has three negatively charged residues (TLEDWTD) [7]. Moreover, the prokaryotic calcium channel Ca_V_Mr (*Meiothermus ruber*) has both glutamic and aspartic (D) acids in the SF: TLEGWVD, with the glycine (G) potentially contributing to Ca^2+^-selectivity [7].

Like bacterial Na_V_s, the C-terminus of the EukCats contains a coiled-coil motif that is hypothesized to be intracellular and perform a range of functions, including stabilizing the quaternary structure of the channel [12], or modulating activation [13] and inactivation [14] of the ion current. Bacterial sodium channels are studied extensively as models for mammalian 4D Na_V_/Ca_V_s, due to their natural variation and relative ease of manipulation [15,16]. The diversity of EukCats provides an expanded library for similar structure-function studies with the advantage of functioning in eukaryotic backgrounds with distinct physiological and metabolic requirements.

Phylogenetically, EukCats group by taxa; the characterized EukCats from diatoms and haptophytes (including coccolithophores) form distinct clades, EukCatA and EukCatB, respectively [3]. We focused on a EukCatA from the diatom *Odontella sinensis* and a EuKCatB from the coccolithophore *Emiliania huxleyi*. *Odontella sinensis* encodes three, single-domain EukCatAs [3] that are functionally non-selective Ca_V_s with ~10-fold faster kinetics than the Na^+^-selective bacterial channel NaChBac [3,6]. The selectivity filter (on the pore loop between S5 and S6) of OsEUKCATA1, from *O. sinensis*, contains two aspartic acid residues (TLDAWAD) that result in a channel with a similar permeability profile as non-selective NaChBac mutants (SF: LEDWAS and LEDWAD) [3,17]. *Emiliania huxleyi* encodes both 4D (two or three) and multiple EukCatB isoforms (three or four), depending on the genetic strain [2]. EukCatB channels are Na^+^-selective, but exhibit dependency on intracellular Ca^2+^, which is potentially regulated through the EF-hand domain on the C-terminus. Their kinetics are ~10-fold faster than those of the EukCatAs, and the characteristic currents generated by EuKCatB channels appear to be very similar to fast metazoan voltage-gated Na^+^ currents [2]. The selectivity filter of the *E. huxleyi* channel that we tested, EhEUKCATB1, contains two glutamic acid residues (TGESWSE) [3], which is similar to bacterial 1D Na^+^-selective channels [6,7].

Marine and terrestrial organisms deploy a wide range of neurotoxins for defense or to capture prey. Some of these toxins specifically target ion channels in the neuromuscular system and are used to characterize the structure and function of ion channels, and identify potential therapeutic drug targets. Conotoxins are soluble peptides produced by marine snails of the genus *Conus*; µ-CTx PIIIA (from the piscivorous *Conus purpurascens*) is injected into prey to rapidly paralyze them before ingestion [18]. µ-CTx PIIIA is a potent antagonist that blocks sodium currents with an IC_50_ of 44 nM and 1 pM for 4D Na_V_s and 1D bacterial Na_V_s, respectively [9,19]. The inhibition of the current is hypothesized to result from the toxin physically occluding the channel pore [8,20]. Multiple conformations of µ-CTx PIIIA-channel binding have been modeled for two 1D bacterial channels, Na_V_Ab (from *Arcobacter butzleri*) [8] and NaChBac [9]. In the majority of models, the negatively charged pore, comprising the four pore-loops of the protein tetramer, is occluded by one of the six positively charged residues of µ-CTx PIIIA, while some of the remaining positively charged residues of the toxin interact with aspartic or glutamic acid in the selectivity filter, S5-P1 linkers (NaChBac) [9], or the outer vestibule (Na_V_Ab) formed by the P2 helices [8].

Brevetoxins are polycyclic ethers produced by the harmful bloom forming dinoflagellate *Karenia brevis* that accumulate in shellfish or are released into the water column when cells lyse [21]. PbTx-2 and PbTx-3 are both found intracellularly and in the extracellular environment [22]. PbTx-2 is the parent compound found in the highest concentrations, while PbTx-3 is a reduced form of PbTx-2 that persists in the water column even after *K. brevis* cell concentrations dissipate. Therefore, PbTx-3 is the compound most frequently used in research [22]. In contrast to µ-CTx PIIIA, PbTx-2 and -3 are potent activators of 4D Na_V_s, Na_V_ 1.2, and Na_V_ 1.4 in animals and humans [23], with IC_50_ in the low nanomolar range [24,25]. The lipophilic brevetoxins span the membrane and interact with 4D Na_V_s in the gap between D1 and DIV where they bind to residues across DI-S6, the intracellular linker to DII, and the extracellular turret loop between DIV-S5 and the P1 helix; these toxin binding regions are collectively known as site 5 [10]. Brevetoxin exposure causes a shift in activation towards more negative membrane potentials and a decrease in the rate of current inactivation leading to prolonged membrane depolarization and repetitive firing of action potentials in animal neurons [24,26]. To date, only one study has explored the impact of brevetoxin on membrane excitability in members of the phytoplankton community [27]; however, brevetoxins have never been tested on 1D channels of any kind before.

Voltage-gated EukCat channels provide a eukaryotic alternative to 1D bacterial channels that have, to date, served as models of the more complex four-domain metazoan channels in structure–function studies. Our goal was to investigate the toxin sensitivity of 1D EukCats to three marine toxins, µ-CTx PIIIA, PbTx-2, and PbTx-3. Phytoplankton are unlikely to encounter µ-CTx PIIIA in the environment; thus, our experiments tested for channel-specific effects of the toxin on function only. Phytoplankton exposed to *K. brevis* experience a range of adverse physiological effects on photosynthesis, membrane integrity, growth, and other metabolic processes [28,29,30]. Non-toxic phytoplankton in *K. brevis* blooms will likely be exposed to brevetoxins, which target Na_V_s in multicellular organisms. Therefore, our objective was to investigate toxin-EukCat interactions for similar targeting in order to assess possible disruption of EukCat-based cell signaling and predict potential outcomes in phytoplankton ecophysiology.

## 2. Results and Discussion

We used whole cell patch clamp electrophysiology to record peak evoked OsEUKCATA1 and EhEUKCATB1 currents in HEK cells. Currents measured from OsEUKCATA1 and EhEUKCATB1 were typical of those previously described (Figures S1 and S2, respectively [2,3]). OsEUKCATA1 currents are smaller and slower to activate, inactivate, and recover from steady state inactivation than EhEUKCATB1 currents (Figures S1 and S2). There is a resemblance between the non-selective OsEUKCATA1 and the Ca^2+^-selective bacterial channel Ca_V_Mr in the magnitude and speed of current activation and inactivation [7], while the large currents and fast kinetics of EhEUKCATB1 are strikingly similar to those of mammalian Na_V_s [31].

**Figure 2:**
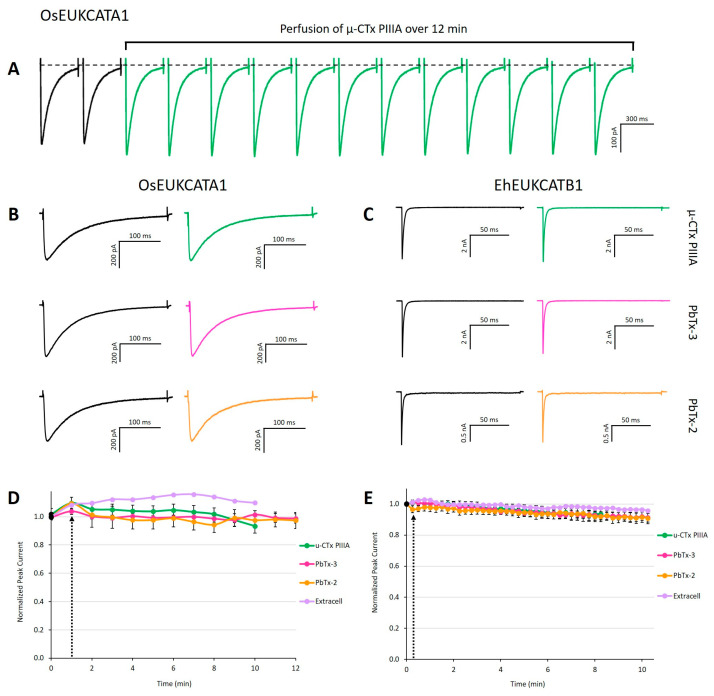
Exposure to µ-CTx PIIIA, PbTx-2, and PbTx-3 does not alter evoked currents. (**A**) Concatenation of peak currents of OsEUKCATA1 in response to a depolarizing voltage pulse from −120 to −10 mV measured every 60 s in the presence of 100 nM µ-CTx PIIIA over a 12 min exposure. Representative peak currents prior to (left traces) and after 12 min exposure to each toxin (right traces) for (**B**) OsEUKCATA1 and (**C**) EhEUKCATB1. (**D**) Peak current (±SE) over time for OsEUKCATA1 and (**E**) EhEUKCATB1 during toxin exposure (10–12 min). Black dot: peak current prior to addition of treatments. Black arrow indicates start of perfusion or bolus addition of treatment. Purple: extracellular solution added via perfusion (*n* = 1). Orange: 500 nM PbTx-2 + DMSO-extracellular (OsEUKCATA1: *n* = 4; EhEUKCATB1: *n* = 6). Pink: 500 nM PbTx-3 + DMSO-extracellular (OsEUKCATA1: *n* = 6; EhEUKCATB1: *n* = 7). Green: 100 nM µ-CTx PIIIA + extracellular-nanopure (OsEUKCATA1: *n* = 10; EhEUKCATB1: *n* = 8). Depolarizing, single-pulse protocols were used to elicit peak currents for OsEUKCATA1 (holding potential: −120 mV; test pulse: −10 mV) and EhEUKCATB1 (holding potential: −130 mV; test pulse: −50 mV).

Peak currents were compared between control and toxin-exposed conditions, in which either toxin or extracellular media were perfused extracellularly. The three toxins (µ-CTx PIIIA, PbTx-2, and PbTx-3) that we tested had no significant effect on the peak evoked currents produced by either of the 1D EukCat channels when compared to control treatments (Figure 2). However, we observed a small (≤10%) decrease in peak currents from the EhEUKCATB1 channels over time (typically ~10–15 min trial) (Figure 2E). Slow but steady declines in current amplitude over the course of a whole cell patch clamp recording are generally attributed to channel run-down caused by the dilution or wash-out of enzymatic cofactors (e.g., ATP and AMP) by the intracellular solution in the patch clamp pipette [32].

**Figure 3:**
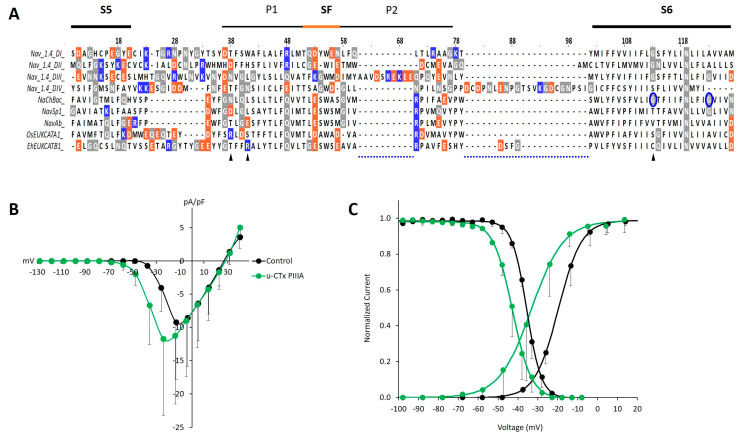
Homology and variation in the S5, S6 linker region of EukCats may underlie the lack of µ-CTx PIIIA block, yet inform effects on activation and steady state inactivation as observed in OsEUKCATA1. (**A**) Multisequence alignment of µ-CTx PIIIA binding region at site-1, including the SF and flanking P1 and P2 helices. Orange: acidic, negatively charged residues. Blue: basic, positively charged residues. Gray: neutrally charged glycine (G), asparagine (N), and glutamine (Q) residues. (**B**) Average (±SD) current-voltage relationships of evoked currents from OsEUKCATA1 [*n* = 6] in control extracellular solution (black) or in the presence of 100 nM µ-CTx PIIIA (green). Currents are normalized to cell capacitance (pA/pF). (**C**) Activation (ascending curves to the right) and steady state inactivation (descending curves to the left) based on average calculated conductance normalized to peak conductance and a two-pulse depolarization protocol, respectively. Curves were fitted with the Boltzmann equation [activation: *n* = 6; inactivation: *n* = 3].

The failure of µ-CTx PIIIA to block evoked currents of either channel was surprising, given the rapid current block (within 3–6 min) observed in bacterial Na_V_s [9], the homology among them and the EukCats across the selectivity filter sequence (Figure 3A). Peak currents were measured in cells held at sufficiently low potentials to avoid steady state inactivation. These results are in stark contrast to the 60% current block observed in NaChBac exposed to µ-CTx PIIIA [9]. The net negative charge and symmetrical distribution of acidic residues within the pores of 4D Na_V_s and 1D BacNa_V_s were hypothesized to facilitate the binding of one of the positively charged residues extending from the main body of µ-CTx PIIIA [8,9,20], suggesting that the similar symmetry and net negative charge of the EukCat pores (−8 compared to −7 in Na_V_1.4) (Figure 3A) would allow for toxin binding. However, the lack of homology, potentially indicating structural variation between the EukCats and the toxin-sensitive 1D bacterial Na_V_s [9] and mammalian target Na_V_1.4 (Figure 3) across the S5-P1 linker [9] and P1 and P2 helices [8] may explain the absence of any direct current block of the EukCat channels.

We next tested the effect of toxins on the kinetics of EukCat currents. However, for the remainder of this paper, we present only data for OsEUKCATA1 due to the large currents (nA) of EhEUKCATB1 that exceeded those conventionally tested using single-electrode patch clamp methods (Figure S3). Series resistance (R_series_) of the electrode introduces a voltage error that is proportional to the currents that flow through the R_series_ of the electrode when recording such large currents. Common remedies, such as two-electrode voltage clamping, were impractical because HEK293 cells are too small, and dynamic R_series_ compensation frequently failed due to oscillations that caused cell instability. While post-hoc corrections are acceptable for smaller currents, because they result in smaller command voltage error, our ability to interpret the very large currents elicited from EhEUKCATB1 using the same approach was hindered. Nevertheless, currents from OsEUKCATA1 were much smaller, and the test voltages were adjusted post-hoc for R_series_ errors.

Peak currents evoked by OsEUKCATA1 are variable in both the control and paired µ-CTx PIIIA conditions, suggesting the inefficiency of protein expression or tetramer assembly from the single subunits in the heterologous system. Although we did not observe a µ-CTx PIIIA-induced block of OsEUKCATA1 currents, the toxin affected the voltage dependency and the kinetic rates of activation and inactivation (Table 1). Exposure to µ-CTx PIIIA caused significant −14 and −7 mV shifts in the voltages of half-activation (V_act_) and half steady state inactivation (V_inact_), respectively, in OsEUKCATA1 (Figure 3B,C; Table 1). The rates of OsEUKCATA1 activation and inactivation, calculated as time constants (τ) of the rising and falling phases of evoked currents, were significantly faster during µ-CTx PIIIA exposure (Table 1). Interestingly, µ-CTx PIIIA exposure of two bacterial Na_V_s (NaChBac and Sp1) also caused negative shifts in the steady state inactivation curves and increased rates (τ) of inactivation for both channels when they were tested at toxin concentrations in which a proportion of the channels was not blocked [9]. Such shifts in the voltage dependency of activation or steady state inactivation may be due to state-dependent binding of the toxin to the channel, as modelled for a µ-CTx PIIIA-bound inactivated channel [9]. Modeling EukCat-toxin binding to inform direct experimental approaches, such as site-directed mutagenesis of both the toxin and the channel, would greatly benefit our understanding of the mechanisms underlying the kinetic effects of µ-CTx PIIIA on OsEUKCATA1.

**Figure 4:**
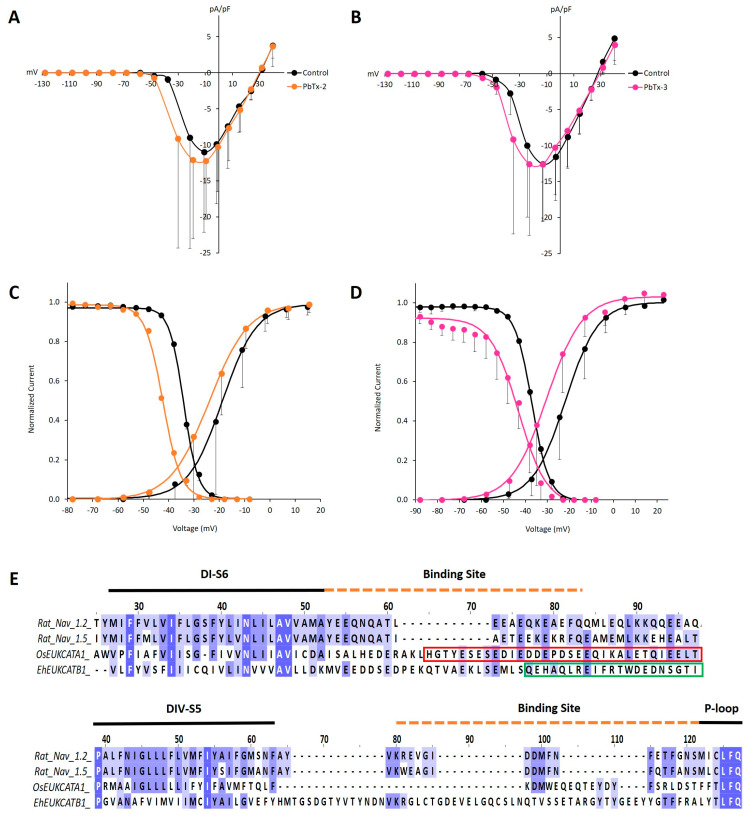
OsEUKCATA1 voltage-dependent activation and steady state inactivation are shifted towards negative voltages when exposed to PbTx-2 and PbTx-3. (**A**) Average (±SD) current–voltage relationship (IV) in control conditions (black) or when exposed to PbTx-2 (orange) (*n* = 5) and (**B**) PbTx-3 (pink) (*n* = 6). Currents are normalized to cell capacitance (pA/pF). (**C**) Voltage-dependent activation (right) (*n* = 5) and steady state inactivation (left) (*n* = 4) in the presence of PbTx-2 (orange) or (**D**) PbTx-3 (pink) [activation: *n* = 6; inactivation: *n* = 5]. Currents were normalized to G_max_ (activation) or I_max_ and averaged (±SD). The curves were fitted (lines) with the Boltzmann equation. Control conditions: extracellular solution +500 nM DMSO. Toxin concentration: 500 nM PbTx-2 or PbTx-3, respectively, dissolved in 500 nM DMSO-extracellular solution. (**E**) Multisequence alignment of rat 4D Na_V_s and EukCats at the PbTx binding receptor, site-5, across DI-S6 (upper) and DIV-S5 (lower). All sequences were trimmed before being aligned separately to show DI and DIV regions. Red box: coiled coil domain of OsEUKCATA1. Green box: EF hand domain of EhEUKCATB1.

Brevetoxins have not been tested directly on 1D channels previously; therefore, our primary point of reference is the increased incidence of action potentials in 4D Na_V_s of neurons and skeletal muscles [24,25,33]. Similar to our observations for µ-CTx PIIIA exposure, we did not observe any change in the magnitude of OsEUKCATA1 and EhEUKCATB1 peak currents in the presence of PbTx-2 or PbTx-3 (Figure 2B–D). Brevetoxin exposure resulted in a statistically significant negative shift (−9 mV) in V_act_ for OsEUKCATA1 with PbTx-3, but not PbTx-2 (Table 1; Figure 4). Unexpectedly, concomitant and statistically significant negative shifts in V_inact_ also occurred in currents from OsEUKCATA1 when exposed to each of the congeners (Table 1; Figure 4C,D). The trains of action potentials that are a hallmark of brevetoxin exposed neurons and muscle cells result from large negative shifts in activation potentials [25,26]. Increased membrane excitability of diatoms could therefore result from negative shifts in V_act_ but may also be countered by similar shifts in V_inact_ in the presence of PbTx-3; a definitive understanding requires further investigation. The two brevetoxins did not affect the rates of activation and inactivation in any clear pattern. The time constant (τ) of inactivation decreased during PbTx-2 exposure (Table 1), while PbTx-3 appeared to affect τ_act_. We view the latter results cautiously, given the rapid rates of activation and the limitations of curve fitting procedures to determine τ_act_.

The relative insensitivity of the EukCats to PbTx is likely attributable to the poor conservation of critical binding regions known to be important in its effect in 4D animal Na_V_s (Figure 3E,F). The toxin is proposed to gain access to 4D Na_V_ channels by integrating into the membrane-filled gap between DI and DIV where it interacts with DI-S6 and the intracellular linker to DII on one side of the gap and DIV-S5 and its extracellular turret loop [10,11] on the other. While 4D Na_V_s contain a single C-terminal with an intracellular regulatory domain [34,35], 1D channels contain four. The intracellular C-termini of 1D subunits are rich in negatively charged acidic residues forming functionally diverse regions that typically, but not exclusively, employ coiled-coil domains. The lack of a PbTx effect on peak EukCat currents may be explained by the relative lack of sequence identity between their S6 regions and the corresponding DI-S6 of 4D Na_V_s. Moreover, the C-terminal intracellular coiled-coil domain of OsEUKCATA1 and the calcium-binding EF-hand domain of EhEUKCATB1 present on each of the individual subunits are not present in 4D Na_V_s (Figure 4E). Similarly, the region spanning DIV-S5 to S6 lacks sequence identity and contains an extraordinarily long extracellular loop extending to the pore domain in EhEUKCATB1 (Figure 4F).

Kitchen et al. (2018) demonstrated that whole-cell currents that underlie action potentials in the diatom *O. sinensis* were partially blocked after exposure to PbTx-3, although the cells experienced little change in membrane potential over time with no obvious hyperexcitability [27]. The reversal potential of the native diatom current also shifted toward more positive voltages in the presence of the toxin, suggesting a change in permeability in favor of Ca^2+^ ions, although there were no changes in the kinetics of native *O. sinensis* currents [27]. In contrast, there was no block or PbTx-induced shift in the reversal potential of the heterologously expressed OsEUKCATA1. Instead, PbTx-3 induced negative shifts in the voltage dependency of activation and steady state inactivation and reduction in the kinetic time constants that were not observed in native diatom currents [27]. Potential explanations for the differences we see between the heterologously expressed OsEUKCATA1 and native *O. sinensis* currents may be attributed to structural requirements of the native channel that are not yet known. Quaternary 4D Na_V_s include the heteromeric α-subunits that form the channels and auxiliary β-subunits that perform a variety of functions and are also targets for toxins [36]. Four-domain Ca_V_s complex with β-subunits in addition to α2δ and γ-subunits in animals, but homologs to these subunits have not been identified in plants with Ca_V_s [37]. No 4D Na_V_s have been identified in the transcriptome of *O. sinensis* [3], so our assumption is that the currents observed in the diatom were from homotetrameric channels, as we also assume that heterologously expressed OsEUKCATA1 are homotetramers. However, three distinct 1D transcripts were identified in *O. sinensis* [3], suggesting that the endogenous channel may be heteromeric. Moreover, while homologs of auxiliary proteins have not been identified in diatoms, we cannot discount possible interactions between the channel and novel auxiliary or regulatory proteins that would modify native channel conductance and kinetics as compared to the heterologous system.

Single-domain voltage gated ion channels are extremely diverse in primary structures and thus provide insight to mechanistic differences in the voltage dependency of gating and kinetic parameters that are tested through electrophysiology, modeling, and crystallographic structures [15]. Although we cannot definitively explain toxin-induced shifts toward more negative voltages in the activation and steady state inactivation curves of OsEUKCATA1, our comparative study of the two EukCat channels in the context of other 1D channels highlights areas for further research. Activation, a response to depolarization of the cell membrane, is sensed via the VSD on S4. Membrane depolarization causes an extracellularly directed shift in S4 [38] that is transferred through S5 to S6 displacing the intracellular ends of S6 and opening the activation gate [39]. Residues within S6 provide flexibility (e.g., through the “glycine hinge”) to open the activation gate in some bacterial Na_V_s; site-directed mutagenesis substituting proline for glycine (G219P; corresponding to Figure 3, site 113) of NaChBac shifted activation to more negative voltages, likely due to stiffening of the helix [40]. The S6 glycine in OsEUKCATA1 (Figure 3A, site 114) suggests that a glycine hinge activation mechanism is also possible in EukCats. Therefore, if toxins bind and stabilize or stiffen the EukCat S6 helix, this will result in negative shifts in activation.

EukCats have much faster rates of inactivation (represented by τ_inact_) than bacterial Na_V_s, but the slower inactivation of the non-selective Na^+^/Ca^2+^ OsEUKCATA1 is similar to slow C-type inactivation achieved through conformational changes within the pore domain (S5, S6) [2,3]. Mechanistically, conformational shifts in the pore region are likely induced by movements of the P1 and P2 helices on the extracellular side of the channel [41], intramembrane movements of the glycine hinge of S6 [40,42], or interactions among the four intracellular C-terminal α-helices that promote closing of the pore [14]. The fact that both hydrophilic µ-CTx PIIIA and hydrophobic brevetoxins caused negative shifts in the steady state inactivation of OsEUKCATA1 suggests that toxin binding may be state-dependent, even if the binding conformation is unique for each class of toxin. The P1 and P2 helices that connect the pore loop to S5 and S6, respectively, are implicated in both µ-CTx PIIIA- and brevetoxin-mediated effects on the steady state inactivation of the channel [8,9,10]. The four P1 helices of 1D channels each span a membrane filled gap between neighboring subunits [41], and their mobility during steady state inactivation may allow brevetoxins to access those gaps and thus the regulatory elements of the channel. The inter-subunit gaps of tetrameric 1D channels are analogous to the gap between DI and DIV of Na_V_s through which brevetoxins access site 5 [10]. Using computational modeling of NaChBac, Finol-Urdaneta et al. (2018) predicted that P2 was displaced by µ-CTx PIIIA in the inactivated state [9]. Notably, both the P1 and P2 helices contain negatively charged amino acids that support hypotheses for binding by µ-CTx PIIIA and blocking of currents [8,9], but those same sites in the EukCats may be engaged alternatively during steady state inactivation-dependent interactions.

Because we are not able to test the effects of negative shifts in the activation and steady state inactivation of 1D channels on membrane excitability in the native diatom, we employed a simple model by Zeberg et al. (2020) [43] that we parameterized with metrics from observed native *O. sinensis* resting potentials and conductance [1] and control and toxin-exposed OsEUKCATA1 activation and steady state inactivation curves. Using this model, we tested a range of current injection stimuli to the model diatom membrane. While Taylor (2009) evoked action potentials from the diatom with a 4 µA cm^−2^ stimulus, a sustained stimulus of 1 µA cm^−2^ was sufficient to evoke a single delayed action potential in the model under control conditions (Figure S4A). Additionally, stimuli of 2 and 5 µA cm^−2^ in control conditions, respectively, produced repetitive action potentials with decreasing magnitude and one single action potential that fired with a shorter delay (Figure S4B,C). Having established membrane excitability with control parameters, we shifted V_act_ and V_inact_ −10 mV to simulate OsEUKCATA1 exposure to µ-CTx PIIIA or PbTx-3. A single action potential fired more quickly than in control conditions for all three stimuli, but subsequent repetitive action potentials evoked by the current injection were damped by the more negative potentials of steady state inactivation (Figure S4). Using this modelled scenario, we predict that in the diatom, the toxin-induced rapid activation of action potentials, combined with fast recovery from steady state inactivation, would result in a higher frequency of action potentials in response to membrane depolarization, potentially leading to hyperexcitability of the diatom membrane with profound impacts on cellular physiology and environmental sensing.

## 3. Materials and Methods

### 3.1. Plasmid Cloning and Purification

Phytoplankton transcript sequences were synthesized (GenScript, Piscataway, NJ, USA) previously in human cell lines through codon-optimization, placing a Kozak sequence (GCCACC) directly upstream of the start codon and removing the stop codon; the DNA fragments were then subcloned into pcDNA3.1-C-eGFP with the GFP fused to the C-terminus of the protein to provide a marker for successful transfection [3]. Larger quantities of each plasmid were produced for experimental purposes by growing them in 50 mL *Escherichia coli* cultures under standard conditions (LB media at 37 °C) and isolating them using a HiSpeed Midi Kit (QIAGEN, Hilden, Germany) according to the manufacturer’s instructions.

### 3.2. HEK293 Cell Culturing

Stock human embryonic kidney (HEK293) cells (ATCC CRL-1573; ATCC, Manassas, Virginia) were cultured in vented T25 flasks (Greiner) in a humidified incubator (37 °C, 5% CO_2_). Cell growth media contained Dulbecco’s modified eagle medium (DMEM) plus Glutamax (Gibco ThermoFisher Scientific, Waltham, MA, USA), 10% fetal bovine serum (FBS) (Gibco ThermoFisher Scientific), penicillin-streptomycin stock (100 µg/mL) (Gibco ThermoFisher Scientific), and normocin (100 µg/mL) (InvivoGen, San Diego, CA, USA). Upon reaching >70% cell confluence (48–72 h), cells were passaged with a dilution factor of 1:6 or 1:12 (cell/mm^2^) using Dulbecco’s Phosphate Buffered Saline (DPBS) (Gibco ThermoFisher Scientific), trypsin EDTA (Gibco ThermoFisher Scientific), and DMEM.

### 3.3. Plasmid Transfection into HEK293 Cells

HEK293 cells were plated on poly-L-lysine (0.01%; Sigma Aldrich, St. Louis, MO, USA) coated glass coverslips (35 mm), one day prior to transfection. Plasmid transfections were prepared using Lipofectamine 2000 (Invitrogen ThermoFisher Scientific) and plasmid DNA (~0.5 µg/µL), each mixed individually with Opti-MEM (Gibco ThermoFisher Scientific). The DNA and lipofectamine were combined and incubated at 37 °C for 5 min before being added to plated cells. Cells were incubated for 4 h, following which the transfection reagents were removed and replaced with fresh growth media. Approximately 24–48 h post-transfection, HEK cells were surveyed for EukCat protein expression using a multiwavelength illuminator (polychrome IV; TILL photonics, Kaufbeuren, Germany) attached to an Olympus IX-71 inverted phase contrast microscope to detect eGFP fluorescence (Ex 490 ± 10 nm, Em 530 ± 20 nm).

### 3.4. HEK293 Whole-Cell Patch Clamp Electrophysiology

Electrophysiological experiments were performed at room temperature on transfected HEK cells using an Axopatch 200b amplifier (Molecular Devices, San Jose, CA, USA) controlled through a Digidata 1440A analog to digital converter using pCLAMP 10.2 software (Molecular Devices) on a PC computer. Membrane voltage and current signals were Bessel filtered at 2KHz and sampled at 5 KHz. Borosilicate glass capillary electrodes (O.D.: 1.5 mm, 0.86 mm) were pulled with a P-97 puller (Sutter Instruments, Novato, CA) to construct patch pipettes (5–8 MΩ resistance) and coated with beeswax to minimize pipette capacitance. Coverslips with adherent cells were placed individually into glass-bottomed petri dishes containing 0.2 μm filtered (cellulose acetate) extracellular solution (140 mM NaCl, 4 mM KCl, 5 mM CaCl_2_, 1 mM MgCl_2_, 10 mM HEPES, 5 mM glucose, 5 mM D-sorbitol; pH 7.4). An intracellular pipette solution (130 mM CsF, 10 mM NaCl, 1 mM MgCl_2_, 1 mM CaCl_2_, 10 mM EGTA, 10 mM HEPES, 5 mM TEA-Cl; pH 7.2) was 0.2 µm filtered (nylon) and dispensed into pulled patch pipettes. Throughout recordings, seal resistance, series resistance, and whole cell capacitance were monitored and recorded. Command voltage was corrected for liquid junction potentials, which were calculated using the LJP-Calculator feature of the pClamp program. Series resistance was corrected by manual subtraction post-recording. Corrected voltages were used to plot current–voltage (IV), activation, and steady state inactivation curves. The peak amplitudes of evoked currents were normalized to cell surface area by dividing the peak current value by cell capacitance (pA/pF). Activation curves were constructed by plotting normalized sodium conductance (G_Na_) as a function of corrected holding voltage and fitted with the Boltzmann function:

I = Gmax (Vm − Vrev)/(1 + exp[(Vm − V_act_)/k]) (1)

In this equation, the peak current (I) is evoked by a specific holding voltage (V_m_), where G_max_ is the maximum slope conductance, V_rev_ is the reversal potential, V_act_ is the voltage that describes the midpoint of the activation curve, and k is the slope factor. Steady state inactivation curves were constructed by plotting normalized peak current (I/I_max_) as a function of corrected prepulse voltage and fitted with the Boltzmann function:

I/Imax = 1/(1 + exp[(Vm − V_inact_)/k]) (2)

In this equation, V_inact_ is the voltage that describes the midpoint of the steady state inactivation curve. Time constants, tau (τ), describe the time for 67% of an asymptotic signal (to or from peak values) to be resolved. Tau of activation (τ_act_) and tau of inactivation (τ_inact_) were fitted with a first order exponential (Chebyshev) and generated from pulse protocols that evoked the peak current (OsEUKCATA1: −10 mV). Tau of recovery from inactivation (τ_recovery_) was acquired from the fitted first order or second order exponential curves (Levenberg–Marquardt) for EhEUKCATB1 and OsEUKCATA1, respectively.

### 3.5. Pharmacological Treatments for Electrophysiological Experiments

Purified PbTx-2 (0.1 mg) and PbTx-3 (0.1 mg), supplied by Dr. J. R. McCall of the University of North Carolina at Wilmington, were prepared as 1 mM stocks dissolved in DMSO and stored at −20 °C. Prior to toxin experiments, intermediate stocks (10 µM) were freshly made up in extracellular solution. Synthetic conotoxin (µ-CTx PIIIA) (Smartox, Saint Egrève, France; Alomone, Jerusalem, Israel; amino acid sequence ZRLCCGFOKSCRSRQCKOHRCC [19]) treatments were prepared as 100 and 20 µM stocks (−20 °C), respectively, in nanopure water and added to the bath to achieve a final concentration of 100 nM. Control treatments for testing the effects of brevetoxin were prepared with DMSO at a 500 nM concentration (0.005% *v*/*v*) in extracellular media (equivalent to the concentration used in PbTx treatments) to compensate for potential effects of DMSO (Figure S5). DMSO and PbTx treatments were applied via bolus addition directly to the bath followed by gentle mixing with the pipette. µ-CTx PIIIA was added by a concentrated bolus delivered and mixed by pipette (*n* = 1) or via a peristaltic pump (at 1.5 mL/min; Minipuls 3, Gilson, Middleton, WI, USA) until the bath (~2 mL) was replaced with toxin solution at the final concentration, at which time the pump was stopped. Kinetic parameters for the two modes of µ-CTx PIIIA were similar. Control voltage clamp protocols (I-V, steady state inactivation, recovery from inactivation) were acquired in the presence of extracellular solution (µ-CTx PIIIA experiments) or DMSO-extracellular (PbTx experiments) conditions, prior to adding toxin treatments. Cells were incubated for 10–15 min after toxin addition. During treatment incubation, a single-pulse voltage clamp protocol, consisting of depolarizing pulses from a holding potential of −130 to −50 mV (EhEUKCATB1) and −120 to −10 mV (OsEUKCATA1) that evoked maximal inward currents, was run to assess changes in peak current in response to treatments.

### 3.6. Statistical Analysis

Averages (mean ± SD or SE) and statistical tests (Student’s paired, two-tail T-test) were computed in Excel. Each replicate (n) represents a single cell and experiment. Statistical significance (*p* ≤ 0.05) was denoted as * *p* ≤ 0.05; ** *p* ≤ 0.005.

### 3.7. Bioinformatic Analysis

Potential mechanistic effects of toxins were assessed using multisequence alignments. Single-domain EukCat sequences from *E. huxleyi* (protein id: CAMPEP_0187645740; MMETSP0994-7) and *O. sinensis* (protein id: CAMPEP_0183296650; MMETSP0160) transcriptomes (MMETSP database) were used as previously described [3]. Multiple sequence alignments of EukCats were performed alongside 4D mammalian Na_V_s [*Homo sapiens* (NCBI; Na_V_ 1.4: NP_000325.4) and *Rattus norvegicus* (NCBI; Na_V_ 1.2: NP_036779.1; Na_V_ 1.5: NP_001153634.1)] and BacNa_V_s [*Bacillus halodurans* (NCBI; BAB05220.1), *Silicibacter ruegeria* (NCBI; AAR26729.1) and *Acrobacter butzleri* (UniProt; A8EVM5_ARCB4)]. Due to numerous non-homologous gapped regions between EukCat and mammalian channels, sequences were trimmed prior to constructing alignments using MAFFT (E-INS-i) version 7 [44] to visualize only potential toxin binding sites and nearby regions. For the analysis of PbTx binding sites, amino acid sequences were trimmed and aligned separately for DI-S6 and DIV-S5. For PbTx binding in DI-S6, all sequences were trimmed 100 residues downstream from the third residue, known as the high field strength site = 0, in the SF. This site most often contains the negatively charged residue that produces the pore motifs of DEKA and EEEE in mammalian Na^+^ and Ca^2+^ channels, respectively. For PbTx binding in DIV-S5, starting from the high field strength site in the SF, all sequences were trimmed 94 residues upstream from that point prior to alignment. The resulting alignment showed receptor site-5, spanning DI and DIV segments, where PbTx is known to interact [10] and their adjacent residues. Amino acid sequences for the analysis of the µ-CTx PIIIA binding site (S5, S6) were trimmed 42 residues upstream and 58 residues downstream from the high field strength site of the SF. The resulting alignment showed adjacent residues of S5 and S6 bordering the P-loop/SF region at site-1 where µ-CTx binding has been modeled to occur [20,45]. Alignments were visualized with JalView version 2.10.4b1 [46] and annotated based on sequence similarity and key residues of interest.

### 3.8. Model of Changes in Membrane Potential

Potential effects of negative shifts of V_act_ and V_inact_ as a result of toxin exposure were assessed using a simple model developed by Zeberg et al. (2020) [43]. Electrophysiological characteristics from the native diatom *O. sinensis* [1] were used to set the following model parameters: (1) a resting membrane potential of −84 mV, (2) an input stimulus of 4 µA cm^−2^, and (3) a slope conductance for sodium of 0.0016 S cm^−2^ (calculated from Figure 2A of Taylor 2009). The surface area of cell membrane used to normalize the latter two parameters was based on cylindrically shaped cells of average size (100 × 30 µm) [1]. We used a general membrane capacitance of 2 µF cm^−2^ and a leak conductance of 0.0005 S cm^−2^, which allowed the model to generate action potentials. Average control and toxin-exposed activation and steady state inactivation curves from this study were used to set V_act_ (control: −21 mV), V_inact_ (control: −37 mV), and the slope factors (activation: 6.8; steady state inactivation 3.8) of the respective curves. The only time constant included is a generalized τ_inact_. The model does not contain further parameterization, for example, for recovery from inactivation.

## 4. Conclusions

Two fundamentally different classes of toxins, µ-CTx PIIIA and brevetoxins PbTx-2 and PbTx-3, similarly affect the Na^+^/Ca^2+^ permeable diatom channel OsEUKCATA1 through voltage dependency of activation and inactivation without affecting peak currents. Peak currents of the Na^+^-selective coccolithophore channel EhEUKCATB1 were similarly unaffected. Our observations with respect to peak currents contrast with the effects that these toxins have on 4D Na_V_s; nevertheless, the differences in conductance and activation and inactivation parameters suggest that the channel selectivity filter and associated P1 and P2 helices are focal points for future research on toxin interactions with animal Na_V_s and EukCats. We present the first observations of brevetoxin exposure on 1D channels, which are especially relevant given that many EukCat-containing marine protist species are present in community assemblages of *Karenia brevis* blooms [47] and that these species may undergo considerable changes in membrane excitability, and thus cellular physiology, upon toxin exposure.

## Figures and Tables

**Table 1:** Kinetic properties of the Na^+^/Ca^2+^ channel OsEUKCATA1 before (control) and after toxin addition. Controls for PbTx were recorded in extracellular media + DMSO. The voltages of half-activation (V_act_) and half steady state inactivation (V_inact_) were determined from voltage dependency curves fit with the Boltzmann equation. Tau of activation (τ_act_) and tau of inactivation (τ_inact_) were determined using peak currents generated from single-pulse protocols (test pulse = −10 mV; holding voltage = −120 mV). Values = mean ± SD. Student’s paired T-tests were used to evaluate statistical significance between control and treatment: * *p* ≤ 0.05; ** *p* ≤ 0.005.

	Control	PbTx-2	Control	PbTx-3	Control	µ-CTx PIIIA
V_act_ (mV)	−18.8 ± 7.7 (*n* = 5)	−23.6 ± 7.8 (*n* = 5)	−21.6 ± 5.7 (*n* = 6)	−30.7 ± 7.5 ** (*n* = 6)	−19.5 ± 2.9 (*n* = 6)	−33.5 ± 9.0 * (*n* = 6)
V_inact_ (mV)	−34.1 ± 0.8 (*n* = 4)	−42.4 ± 1.5 ** (*n* = 4)	−37.0 ± 2.7 (*n* = 5)	−44.6 ± 6.4 * (*n* = 5)	−35.5 ± 1.1 (*n* = 3)	−42.6 ± 3.0 * (*n* = 3)
τ_act_ (ms)	1.7 ± 0.9 (*n* = 4)	1.6 ± 0.9 (*n* = 4)	1.3 ± 0.7 (*n* = 6)	1.1 ± 0.6 * (*n* = 6)	2.0 ± 1.1 (*n* = 10)	1.4 ± 0.9 * (*n* = 10)
τ_inact_ (ms)	51.5 ± 14.3 (*n* = 4)	48.5 ± 13.2 * (*n* = 4)	54.4 ± 7.7 (*n* = 6)	50.0 ± 9.9 (*n* = 6)	62.6 ± 24.5 (*n* = 10)	55.0 ± 20.0 * (*n* = 10)

## Data Availability

The data presented in this study are openly available on figshare, doi:10.6084/m9.figshare.13412714.

## Supplementary Materials

The following are available online at https://www.mdpi.com/1660-3397/19/3/140/s1, Figure S1: Characteristic currents of OsEUKCATA1 from the diatom *Odontella sinensis*; Figure S2: Characteristic currents of EhEUKCATB1 from the coccolithophore *Emiliania huxleyi*. Figure S3: Series resistance (Rseries) of the electrode introduces a voltage error that is proportional to the currents flowing through Rseries of the electrode when recording such large currents in EhEUKCATB1. Figure S4: Negative shifts in activation and inactivation curves result in early firing of the initial action potential under modeled [39] control conditions (black) and toxin exposure (orange). The model was parameterized with characteristics of the native diatom *O. sinensis* or OsEUKCATA1 depending on available data (see Materials and Methods). Control scenarios were modelled with Vact of −21 mV and Vinact of −37 mV. Vact and Vinact were shifted −10 mV for toxin treatments. The only time constant included was a generalized τ_inact_. The model did not contain any parameterization for recovery from inactivation. A single sustained input stimulus was applied to all treatments for the 100 ms duration of model runs. Figure S5: DMSO affects activation and inactivation in OsEUKCATA1. All control treatments of for toxin-exposed comparisons contained DMSO to control for its effects.
