# Supplementary material for: Brevetoxin and Conotoxin Interactions with Single-Domain Voltage-Gated Sodium Channels from a Diatom and Coccolithophore
Source: Mar Drugs. 2021 Mar 2;19(3):140. doi: 10.3390/md19030140 (PMC8002053; doi:10.3390/md19030140)
Supplement: Supplementary file 1 [file marinedrugs-19-00140-s001.pdf]

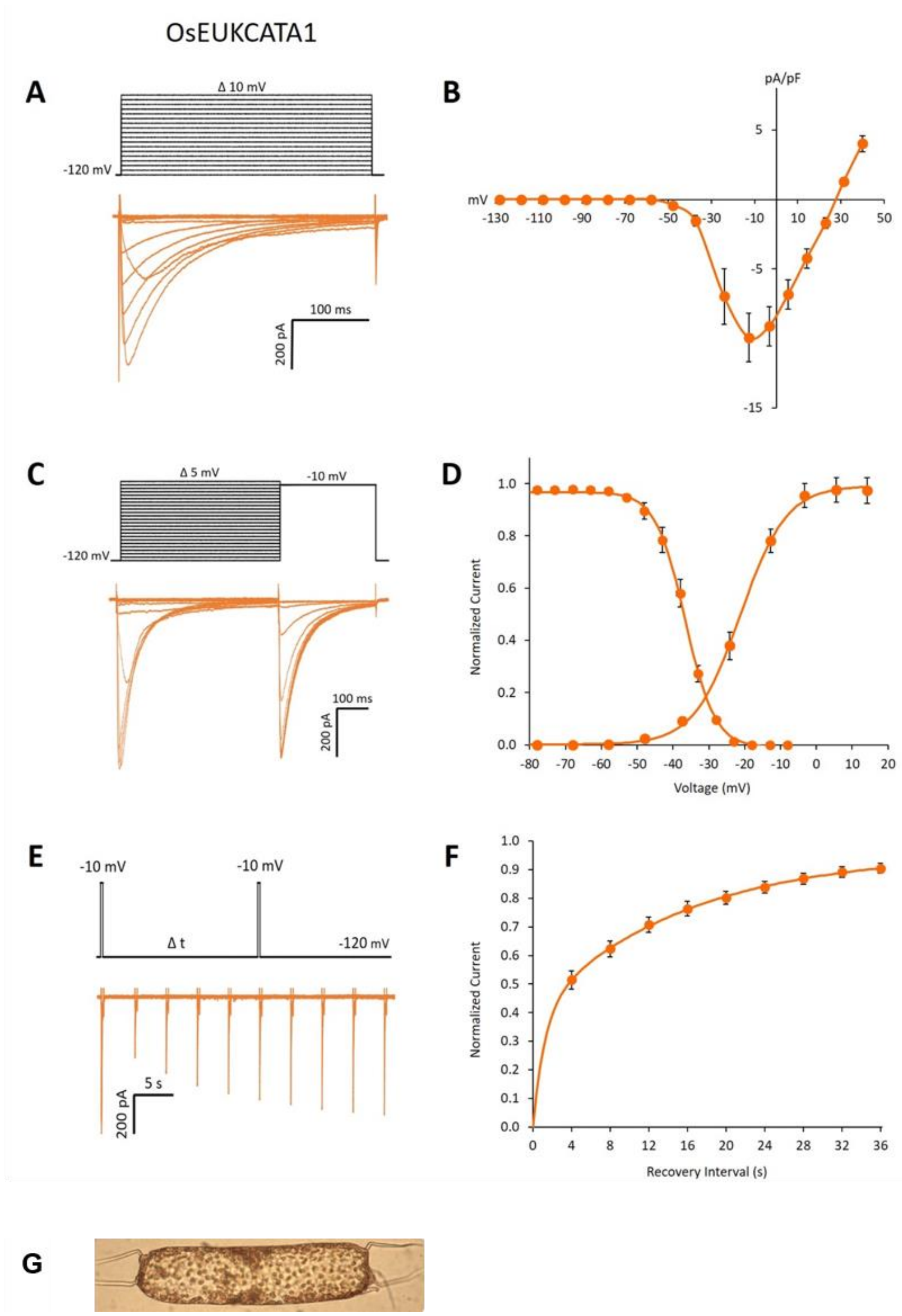

Figure S1. Characteristic currents of OsEUKCATA1 from the diatom *Odontella sinensis*. A) Voltage protocol (upper) used to evoke typical currents (lower) in response to membrane depolarization. B) Average (± SE) current-voltage (IV) curves (right) where current is normalized to cell capacitance (pA/pF). Peak current = -9.9 ± 1.8 pA/pF (n=21). C) Steady-state inactivation protocols (upper) and example current traces (lower) were used to construct D) average (± SE) steady-state inactivation curves (right panel-left side curve). Currents elicited by depolarization (e.g. as in A & D) were used to construct activation curves (right side curve of D). Average normalized data for activation and steady-state inactivation were fitted with the Boltzmann equation. V_{inact} = -37.0 ± 0.9 mV (n=17). V_{act} = -21.3 ± 1.1 mV (n=21). Tau values were acquired from single-pulse protocols. τ activation = 1.8 ± 0.2 ms (n=19); τ inactivation = 59.5 ± 4.4 ms (n=19). E) Voltage protocol (upper) and example current traces (lower) used to measure recovery from inactivation with example current traces were used to construct F) the curve for recovery from inactivation. Average (± SE) normalized data was fitted with a second order exponential using zero as the first coordinates. τ_{re}= 20.3 ± 3.3 s and 1.3 ± 0.2 s (n=16); G) light micrograph of the diatom *Odontella sinensis*, which encodes OsEUKCATA1.

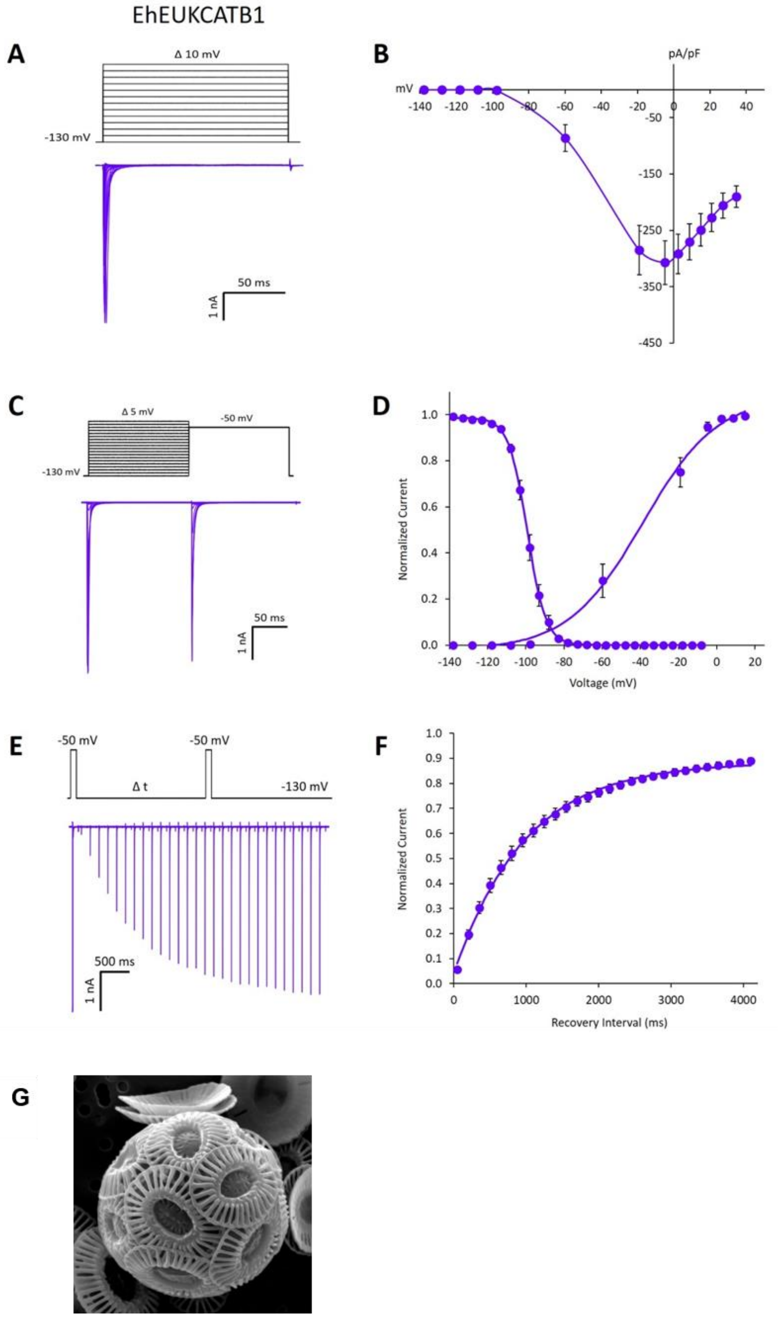

Figure S2. Characteristic currents of EhEUKCATB1 from the coccolithophore *Emiliania huxleyi*. A) Voltage protocol (upper) used to evoke typical currents (lower) in response to membrane depolarization. B) Average (± SE) current-voltage (IV) curves where current is normalized to cell capacitance (pA/pF). Peak current = -306.9 ± 38.8 pA/pF (n=26). C) Steady-state inactivation protocols (upper) and example current traces (lower) were used to construct D) average (± SE) steady-state inactivation curves (E, left curve). Currents elicited by depolarization (e.g. as in A & D) were used to construct activation curves (D, right curve). Average normalized data for activation and steady-state inactivation were fitted with the Boltzmann equation. V_{inact} = -98.8 ± 1.0 mV (n=24). V_{act} = -38.6 ± 4.6 mV (n=26). Tau values were acquired from single-pulse protocols. τ activation = 0.2 ± 0.02 ms (n=22); τ inactivation = 1.5 ± 0.1 ms (n=22). E) Voltage protocol (upper) and example current traces (lower) used to measure recovery from inactivation with example current traces were used to construct F) the curve for recovery from inactivation. Average (± SE) normalized data was fitted with a first order exponential. $\tau_{recovery}$ = 1029.6 ± 116.8 ms (n=14); G) electron micrograph of Emiliania huxleyi, which encodes EhEUKCATB1.

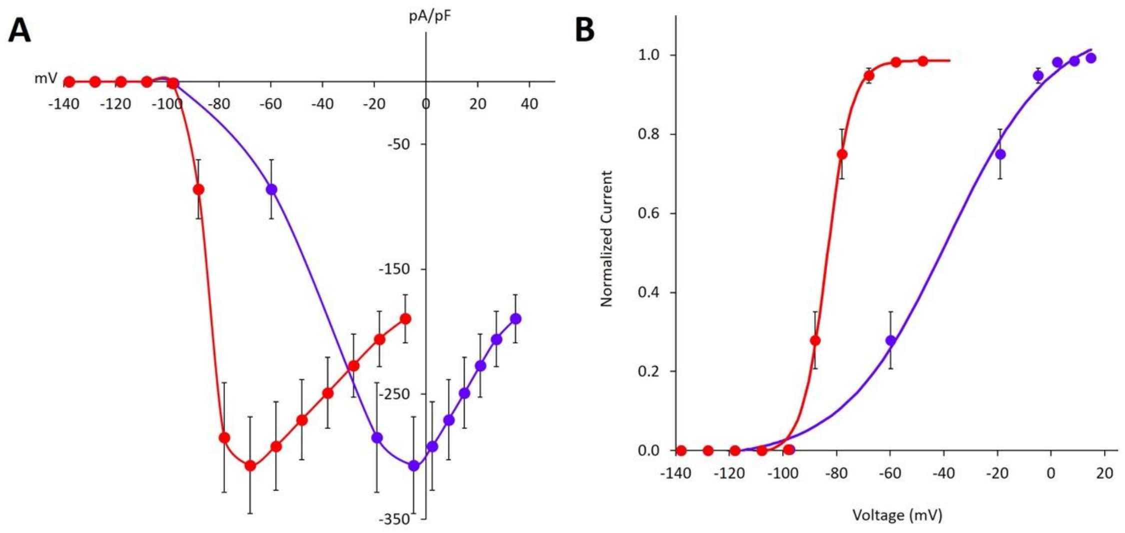

Figure S3. Series resistance (R_{series}) of the electrode introduces a voltage error that is proportional to the currents flowing through R_{series} of the electrode when recording such large currents in EhEUKCATB1. A) IV with (purple) and without (red) voltage correction for R_{series} (n=26). B) Activation curves with (purple) and without (red) voltage correction for R_{series} were fitted with the Boltzmann equation (n=26). Corrections for liquid junction potential were made in all cases.

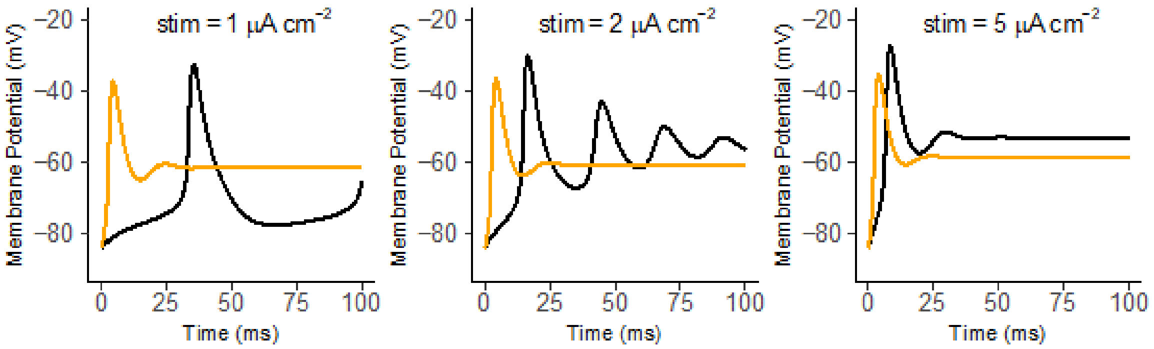

Figure S4. Negative shifts in activation and inactivation curves result in early firing of the initial action potential under modeled [39] control conditions (black) and toxin exposure (orange). The model was parameterized with characteristics of the native diatom *O. sinensis* or OsEUKCATA1 depending on available data (see Materials and Methods). Control scenarios were modelled with V_{act} of -21 mV and V_{inact} of -37 mV. V_{act} and V_{inact} were shifted -10 mV for toxin treatments. The only time constant included was a generalized τ_{inact}. The model did not contain any parameterization for recovery from inactivation. A single sustained input stimulus was applied to all treatments for the 100 ms duration of model runs. A) The 1 μA cm^{-2} input stimulus was the threshold required to evoke an action potential in control conditions; the time course of the action potential, with the 25 ms delay, was also the most similar to those observed by Taylor (2009) in the native diatom *O. sinensis* using a 4 μA cm^{-2} stimulus. Under toxin exposure (inducing a negative shift in V_{act} and V_{inact}), the first action potential fired within ~5 ms because activating voltages were more negative. A second action potential began to form, but inactivation also shifted negatively and damped it. B) At a higher input stimulus (2 μA cm^{-2}), the action potential fired more quickly and repeated action potentials occurred because depolarization of the membrane was maintained outside the range of inactivating voltages. However, in the presence of toxin, the negative shift of activation allowed for early firing of the first action potential, but further action potentials were inhibited by inactivation. C) The greatest stimulus (5 μA cm^{-2}) most closely simulated the stimulus used by Taylor (2009) and allowed for the most rapid action potential under control parameters; however, it was still delayed relative to the action potential generated in the toxin treatment.

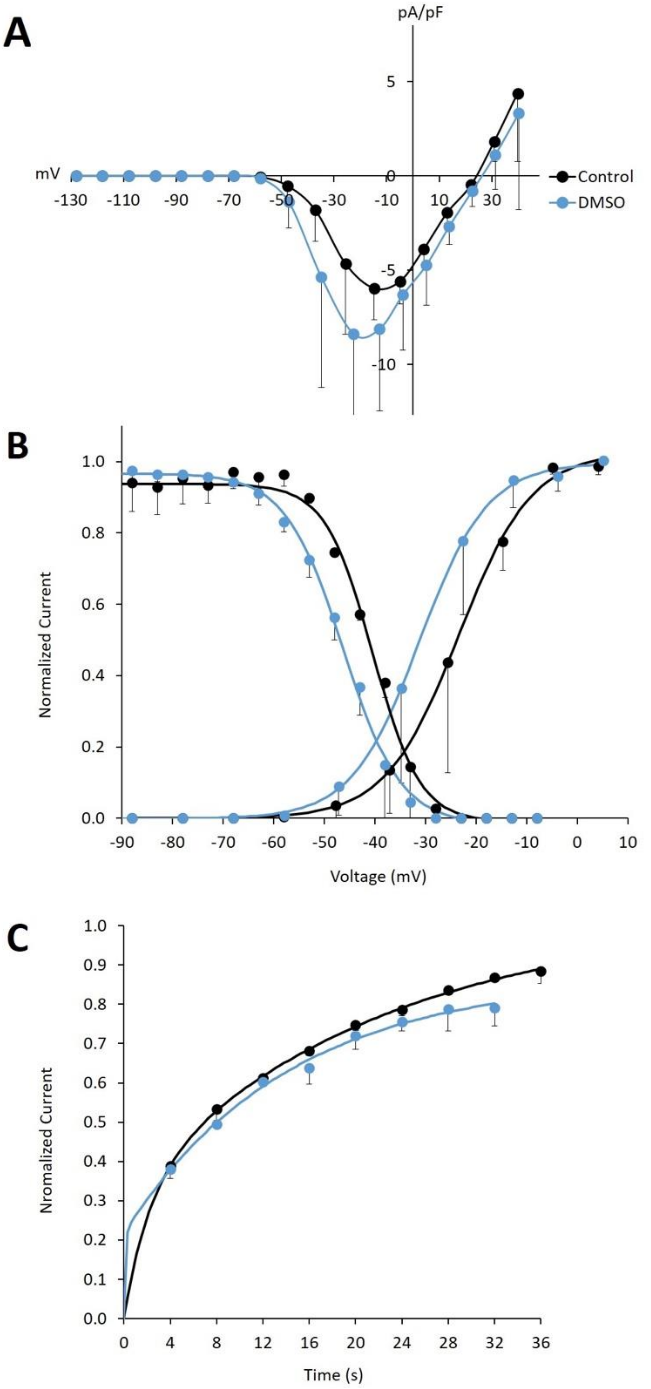

Figure S5. DMSO affects activation and inactivation in OsEUKCATA1. All control treatments of for toxin-exposed comparisons contained DMSO to control for its effects. A) Average (± SD) IV curves with the current normalized to cell capacitance (pA/pF) [n=3], B) Voltage dependency of activation [n=3] and inactivation [n=3]. Average (± SD) normalized data for activation (right side) and steady-state inactivation (left side) curves were fitted with the Boltzmann equation. Significant differences were found in the voltage of half-activation between control conditions (-24.6 ± 6.8) and exposure to DMSO (-31.3 ± 7.1; $p < 0.05$) and the voltage of half-inactivation between control conditions (40.9 ± 6.3) and exposure to DMSO (46.8 ± 7.2; $p < 0.05$). C) Recovery from inactivation. Average normalized data for recovery from inactivation was fitted with a second order exponential for OsEUKCATA1 [n=2]. The presence of DMSO did not affect $\tau_{recovery}$ (15.6 ± 1.6 s and 1.5 ± 1.1 s) when compared to control conditions (25.6 ± 10.2 s and 2.3 ± 1.1 s). Black: Extracellular solution only. Blue: Extracellular solution + 500 nM DMSO (represents 0.005% v/v solution).
